# Molecular Targets for Biological Therapies of Severe Asthma

**DOI:** 10.3389/fimmu.2020.603312

**Published:** 2020-11-30

**Authors:** Corrado Pelaia, Claudia Crimi, Alessandro Vatrella, Caterina Tinello, Rosa Terracciano, Girolamo Pelaia

**Affiliations:** ^1^ Respiratory Medicine Unit, University “Magna Græcia” of Catanzaro, Catanzaro, Italy; ^2^ Department of Clinical and Experimental Medicine, University of Catania, Catania, Italy; ^3^ Department of Medicine, Surgery and Dentistry, University of Salerno, Salerno, Italy; ^4^ Pediatrics Unit, Provincial Outpatient Center of Catanzaro, Catanzaro, Italy; ^5^ Department of Experimental and Clinical Medicine, University “Magna Græcia” of Catanzaro, Catanzaro, Italy; ^6^ Department of Health Sciences, University “Magna Græcia” of Catanzaro, Catanzaro, Italy

**Keywords:** T2-high asthma, IgE, IL-4, IL-5, IL-13, monoclonal antibodies

## Abstract

Asthma is a heterogeneous respiratory disease characterized by usually reversible bronchial obstruction, which is clinically expressed by different phenotypes driven by complex pathobiological mechanisms (endotypes). Within this context, during the last years several molecular effectors and signalling pathways have emerged as suitable targets for biological therapies of severe asthma, refractory to standard treatments. Indeed, various therapeutic antibodies currently allow to intercept at different levels the chain of pathogenic events leading to type 2 (T2) airway inflammation. In addition to pro-allergic immunoglobulin E (IgE), that chronologically represents the first molecule against which an anti-asthma monoclonal antibody (omalizumab) was developed, today other targets are successfully exploited by biological treatments of severe asthma. In particular, pro-eosinophilic interleukin 5 (IL-5) can be targeted by mepolizumab or reslizumab, whereas benralizumab is a selective blocker of IL-5 receptor. Moreover, dupilumab behaves as a dual receptor antagonist of pleiotropic interleukins 4 (IL-4) and 13 (IL-13). Besides these drugs that are already available in medical practice, other biologics are under clinical development such as those targeting innate cytokines, also including the alarmin thymic stromal lymphopoietin (TSLP), which plays a key role in the pathogenesis of type 2 asthma. Therefore, ongoing and future biological therapies are significantly changing the global scenario of severe asthma management. These new therapeutic options make it possible to implement phenotype/endotype-specific treatments, that are delineating personalized approaches precisely addressing the individual traits of asthma pathobiology. Such tailored strategies are thus allowing to successfully target the immune-inflammatory responses underlying uncontrolled T2-high asthma.

## Introduction

Asthma is a very diffuse chronic respiratory disease whose main pathologic features include airway inflammation and remodelling, which are responsible for variable airflow limitation and bronchial hyperresponsiveness (1–3). More than 300 million people currently suffer from asthma worldwide, and this number is probably destined to undergo further increases during the next years (4, 5). In most subjects with asthma a good disease control can be achieved using standard inhaled treatments. However, about 5–10% of patients included in the global population of asthmatic individuals experience various subtypes of inadequately controlled and difficult-to-treat asthma (6). In this regard, severe asthma was jointly defined by both European Respiratory Society (ERS) and American Thoracic Society (ATS) as a condition controlled by high dosages of inhaled corticosteroids (ICS)/long-acting β_2_-adrenergic agonists (LABA) combinations, which can also require the addition of other drugs (i.e. tiotropium, leukotriene modifiers, oral corticosteroids); even worse, severe asthma might remain uncontrolled despite such massive inhaled and systemic treatments (7). Hence, within the overall spectrum of subjects with asthma, severe asthmatic patients are characterized by the most urgent unmet medical needs and can be eligible to add-on biological therapies (8). The latter mainly consist of already licensed monoclonal antibodies targeting specific molecules involved in the pathobiology of type 2 (T2-high) eosinophilic, allergic and non-allergic asthma, including immunoglobulins E (IgE), interleukin-5 (IL-5) and its receptor, as well as interleukin-4 (IL-4) receptor (9–11). Other experimental biologics target upstream innate cytokines such as thymic stromal lymphopoietin (TSLP) (12, 13). Conversely, current pharmacotherapeutic pipelines are very scarce with regard to investigational drugs directed against molecular targets implicated in the pathogenesis of T2-low, mostly neutrophilic severe asthma. Therefore, a careful characterization of the biological mechanisms (endotypes) underlying the different phenotypes plays a key role in driving the clinical choice of the most appropriate add-on therapy for each individual patient with severe asthma (14, 15).

On the basis of the above considerations, the aim of this review article is to outline the cellular and molecular pathophysiology of severe asthma, in order to provide a logical premise for the subsequent discussion of the current and future biological strategies that can be used to treat the patients with uncontrolled disease.

## Pathobiology of Severe Asthma

Asthma is a heterogeneous disease, originating from complex interactions between genetic and environmental factors, which consists of several different phenotypes sustained by cytokine-based biological mechanisms known as endotypes (16, 17). The inflammatory endotypes include eosinophilic, neutrophilic, mixed and paucigranulocitic cellular patterns (2, 18–22). In particular, T2-high eosinophilic inflammation is quite common in patients with either allergic or non-allergic asthma, and can frequently characterize severe and fatal disease (23–27).

T2-high eosinophilic allergic asthma, occurring especially in children and adolescents, develops as a consequence of an intricate cross-talk between innate and adaptive immune responses (28). In particular, allergic asthma is triggered by dust mites, tree pollen and animal dander, which within the airways are captured by dendritic cells that internalize and process these aeroallergens, and also transport them to thoracic lymph nodes. Here, dendritic cells expose on their surface the processed allergen peptides and, within the context of specific HLA class II molecules of the major histocompatibility complex (MHC class II), operate antigen presentation to the T-cell receptors of naïve CD4^+^ T lymphocytes, thus inducing their polarization towards the T helper 2 (Th2) lineage (1). This event is driven by interleukin-4 (IL-4) produced by mast cells and basophils, and is also dependent on selective reciprocal recognition of specific co-stimulatory molecules located on the plasma membranes of dendritic cells (CD80/B7.1, CD86/B7.2, OX40 ligand, ICOS ligand) and T lymphocytes (CD28, OX40, ICOS), respectively (29, 30). As a result of such a complex process of differentiation and activation, mature Th2 cells secrete large quantities of IL-4, IL-13, and IL-5. IL-13 and especially IL-4 induce Ig class switching by stimulating B lymphocytes to synthesize allergen-specific immunoglobulins E (IgE), which bind to high-affinity (FcϵRI) and low-affinity (CD23/FcϵRII) receptors present on both immune/inflammatory and structural cells of the respiratory tract (31–34). These adaptive immune pathways are crucially integrated by innate immune mechanisms involving important functions of airway epithelial cells and innate lymphoid cells, as well as further contributions of dendritic cells (35, 36). Indeed, aeroallergens, respiratory viruses, cigarette smoking and airborne pollutants induce bronchial epithelial cells to produce the innate cytokines thymic stromal lymphopoietin (TSLP), interleukin-25 (IL-25) and interleukin-33 (IL-33), that in turn potentiate Th2-mediated adaptive immune responses and promote the release of IL-4, IL-13, and IL-5 from Th2 lymphocytes and group 2 innate lymphoid cells (ILC2) (37). Another relevant cellular source of IL-4 is represented by T follicular helper cells (Tfh), whose development in lung-draining lymph nodes depends on TSLP-induced activation of dendritic cells expressing OX40 ligand (38). Dendritic cells also secrete CCL17 and CCL22 chemokines, that selectively interact with CCR4 receptors expressed by mature Th2 lymphocytes, thus promoting their migration from thoracic lymph nodes to the airways (39).

In regard to the functions of Th2 cytokines, IL-4 drives IgE biosynthesis, IL-13 mainly contributes to mucus production, airway remodelling and bronchial hyperresponsiveness, and IL-5 is the key inducer of eosinophil differentiation, activation and survival (17, 40). In addition to Th2 lymphocytes, IL-5 is also produced by mast cells, natural killer T cells, eosinophils themselves and especially ILC2, the latter being the main cellular orchestrators of non-allergic eosinophilic asthma (40–42), frequently characterized by a late onset in adulthood. IL-5 is responsible for eosinophil maturation, and in asthmatic patients this eosinophilopoietic action occurs not only in the bone marrow, but also in bronchial mucosa (43–45). Indeed, IL-5 concentrations and the numbers of both mature eosinophils and eosinophil progenitors are increased in induced sputum from asthmatic subjects. High IL-5 levels can be also detected in serum, especially when obtained from patients with severe asthma (46). Moreover, IL-5 exerts an inhibitory effect on eosinophil apoptosis, and the numbers of apoptotic eosinophils are negatively correlated with sputum IL-5 concentrations in stable asthma, as well as during disease exacerbations (47, 48). IL-5 also contributes to eosinophil recruitment within asthmatic airways, thereby cooperating with eosinophil chemoattractants such as eotaxins (49). Furthermore, in patients with T2-high asthma IL-5 stimulates the interaction of eosinophils with periostin, an extracellular matrix protein whose expression resulted to be up-regulated during eosinophil migration towards airways (50). Especially in severe asthma, IL-5-activated eosinophils also contribute to bronchial structural changes *via* the release of powerful pro-remodelling mediators such as transforming growth factor-β (TGF-β) (51, 52).

T2-low asthma is often characterized by airway neutrophilia, particularly in patients suffering from severe disease forms (53). In this regard, the Th17 subset of CD4^+^ T lymphocytes seems to play a pivotal pathogenic role (54–56). Th17 cells produce IL-17A and IL-17F, whose expression was shown to be significantly up-regulated in bronchial biopsies from patients with severe asthma (57). Th17 cell development depends on the coordinate actions of IL-1β, IL-6, and TGF-β, which are essential for induction of differentiation of this cellular immunophenotype (58–60). In addition, IL-21 produced by Th17 cells themselves plays a key role as autocrine amplifier of Th17 response (60, 61). Mature Th17 lymphocytes also express the specific receptor of IL-23, a cytokine which is required to stabilize the Th17 phenotype and to maintain Th17 cells in a state of effective activation (60–62). Cigarette smoke and diesel exhaust particles can induce airway neutrophilia, which was shown to be associated with Th17-dependent severe asthma (63, 64). Infectious agents also seem to be able to trigger Th17-mediated severe asthma, and this effect may involve the assembly of the inflammasome, an intracellular multiprotein complex which activates caspase-1, a protease that converts pro-IL-1β in its active form, thus enabling it to induce Th17 cell differentiation (65, 66). Inflammasome activation was also shown to be implicated in obesity-associated bronchial hyperresponsiveness (67). In severe asthma, Th17 cell polarization and neutrophilic airway inflammation can also be promoted by neutrophil extracellular traps (NETs), consisting of anti-microbial complexes of extracellular DNA, histones and granular proteins extruded from neutrophils that become anuclear cells known as cytoplasts (68–70). In addition to Th17 lymphocytes, other cellular sources of IL-17 include invariant NK T cells, γδ T cells, cytotoxic T cells, and especially group 3 innate lymphoid cells (ILC3) (71, 72). High numbers of ILC3 were found in bronchoalveolar lavage fluid (BALF) from adults with severe asthma, as well as in blood of obese asthmatic children (72, 73). Once secreted from Th17 lymphocytes, ILC3 and other cell types, IL-17A and IL-17F induce bronchial epithelial cells and sub-epithelial airway fibroblasts to release potent neutrophil chemoattractants including IL-8 (CXCL8) and CXCL1/GRO-α (74–76]. IL-17-mediated neutrophilic asthma is often associated with a relevant insensitivity to the therapeutic actions of corticosteroids, which indeed exert an anti-apoptotic effect on neutrophils, thus prolonging their survival (77). In addition to Th17 lymphocytes, also IL-12-dependent Th1 cells can contribute to the pathobiology of severe neutrophilic asthma (59, 78). In fact, Th1 lymphocyte numbers and the levels of their cytokines such as interferon-γ (IFN-γ) and tumor necrosis factor-α (TNF-α) are enhanced in severe asthmatic patients (59, 79).

The mixed eosinophilic/neutrophilic inflammatory phenotype is often associated with severe asthma. In particular, circulating Th2/Th17 cell clones producing both IL-4 and IL-17A were found in asthmatic patients (80). Moreover, high numbers of dual-positive Th2/Th17 lymphocytes secreting large quantities of IL-4 and IL-17 were detected in BALF from patients with severe asthma (81). Indeed, these BALF lymphocytes were shown to concomitantly express two transcription factors such as GATA3 and RORγt (81), which are essential for differentiation of Th2 and Th17 cells, respectively. Such observations corroborated the results of previous studies performed in mice, which had demonstrated that Th2/Th17 lymphocytes were involved in the induction of severe forms of experimental asthma (82). Hence, additional studies are needed to further characterize the cellular phenotypes of dual Th2/Th17 lymphocyte subsets, and to better understand if IL-4 and IL-17 produced by these cells could eventually exert additive or synergistic effects, especially in the pathobiology of severe asthma (83).

In addition to eosinophilic, neutrophilic, and mixed granulocytic inflammatory profiles, also paucigranulocytic histopathological patterns have been found in airway biopsies from asthmatic patients (84, 85). The cellular pathophysiology of this particular asthmatic phenotype, characterized by the lack of increased counts of eosinophils or neutrophils in either sputum or blood, has not been clearly elucidated. However, it appears that paucigranulocytic asthma is featured by an uncoupling of bronchial obstruction from inflammation, possibly due to structural changes mainly resulting in non-inflammatory thickening of airway smooth muscle layer (86).

## Licensed Biological Therapies of Severe Asthma

**Figure 1:**
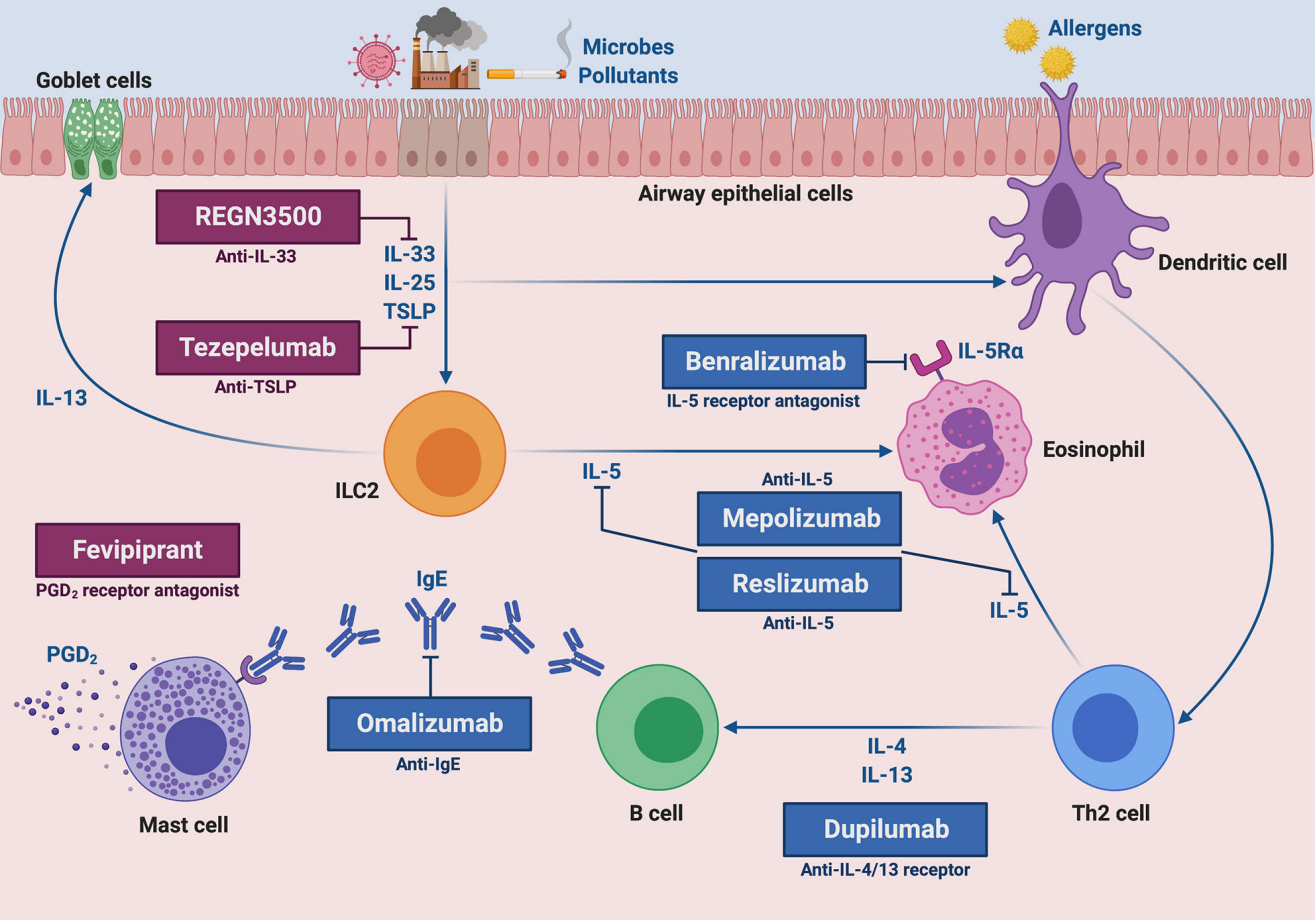
Molecular targets of current and future biological therapies of severe type 2 asthma. The targets of approved add-on biologic treatments (highlighted in blue color) of severe asthma include IgE (omalizumab), IL-5 (mepolizumab and reslizumab), IL-5 receptor (benralizumab), and IL-4/IL-13 receptor complex (dupilumab). Moreover, experimental drugs (highlighted in dark magenta color) such as tezepelumab, REGN3500 and fevipiprant target TSLP, IL-33 and the CRTH2 receptor of PGD_2_, respectively. This original figure was created by the authors using “BioRender.com”.

There are currently five approved monoclonal antibodies for add-on biologic treatment of severe asthma. They include omalizumab, mepolizumab, reslizumab, benralizumab, and dupilumab (**Figure 1**).

Omalizumab has been the first licensed biologic drug for clinical use in the management of severe asthma. This recombinant humanized monoclonal antibody, originally developed in mice, binds to the two Cϵ3 domains of the constant portion of IgE, thus forming IgE/anti-IgE immune complexes that prevent IgE interactions with both high-affinity FcϵRI and low-affinity FcϵRII/CD23 membrane receptors (87, 88). As a consequence, omalizumab inhibits all IgE-dependent cellular and molecular events involved in the immune/inflammatory cascade underlying allergic asthma. Systematic reviews and pooled analyses of randomized controlled trials have clearly shown that omalizumab was able to significantly decrease the rate of asthma exacerbations, and this therapeutic effect was observed up to 48–60 weeks of treatment (89, 90). Such a favourable clinical outcome has been further corroborated by several worldwide real-life studies (91). In addition to confirming the positive impact of omalizumab on asthma exacerbations, emergency room accesses and hospitalizations, real-world experiences have also demonstrated relevant improvements in symptom control, quality of life, and intake of oral corticosteroids (OCS), as well as a lower loss of working and school days (91–93). Despite some discordant published data regarding the effects of omalizumab on lung function (15), many real-life studies have shown that this anti-IgE monoclonal antibody can induce significant and persistent increases in forced expiratory volume in the first second (FEV_1_), lasting 5, 7, and even 9 years (94–96). Moreover, it was also recently reported that omalizumab can effectively improve both clinical manifestations and computed tomography (CT) images of nasal polyps associated with severe allergic asthma (97). All these beneficial outcomes achieved by patients undergoing add-on therapy with omalizumab explain the high degree of adherence to this biologic drug (98). The real-life therapeutic effectiveness of omalizumab coexists with a long-term, very good safety and tolerability profile (99).

Mepolizumab is a humanized IgG1/κ monoclonal antibody of murine origin which binds with high affinity to human IL-5, thus preventing its interaction with the α subunit of IL-5 receptor (IL-5Rα) (100). The efficacy of mepolizumab was firstly evidenced by Nair et al. and by Haldar et al., who showed in a few frequent exacerbators with severe eosinophilic asthma that this biologic drug significantly reduced disease exacerbations, as well as blood and sputum eosinophils (101, 102). These positive effects of mepolizumab were later confirmed by the phase IIb/III DREAM (Dose Ranging Efficacy And safety with Mepolizumab) trial, carried out by Pavord et al. in a much larger number of patients (103). Moreover, MENSA (MEpolizumab as adjunctive therapy iN patients with Severe Asthma) and SIRIUS (SteroId ReductIon with mepolizUmab Study) trials, performed by Ortega et al., and Bel et al., respectively, documented that in subjects with severe eosinophilic asthma mepolizumab lowered asthma exacerbation rate, improved quality of life and symptom control, and also slightly increased FEV_1_ (104, 105). In addition, the SIRIUS study demonstrated that mepolizumab exerted an effective OCS sparing action, thereby decreasing prednisone intake by 50% (105). Furthermore, the phase IIIb MUSCA trial, carried out by Chupp et al., corroborated the significant results achieved by patients undergoing add-on mepolizumab therapy with regard to improvement of health-related quality of life (106). All these randomized controlled studies also reported a very good pattern of drug safety and tolerability. Besides such controlled trials, uncontrolled, open-label, and real-life investigations are providing further information about the therapeutic properties of mepolizumab. Indeed, some real-world data suggest that in clinical practice mepolizumab effectiveness can be even greater than that one observed in randomized controlled trials, and these better results might be dependent on the higher numbers of baseline blood eosinophils detectable in patients enrolled in real-life experiences (107, 108). The latter have shown that mepolizumab is very effective in both non allergic and allergic patients with severe eosinophilic asthma, also in case of switching from omalizumab to mepolizumab because of an inadequate disease control provided by anti-IgE treatment (109–111). With regard to lung function, it is noteworthy that in real-life setting mepolizumab was able not only to increase FEV_1_, but also to improve airflow limitation at level of small airways (112). Mepolizumab was capable of inducing beneficial therapeutic effects also in severe nasal polyposis, thus improving subjective symptoms and endoscopic nasal polyp score, as well as leading to a decreased need for surgical polypectomy (113).

Another anti-IL-5 biologic drug is reslizumab, a humanized IgG4/κ monoclonal antibody of rat origin, whose clinical and functional effects have been assessed in many randomized trials (114, 115). The first phase II trial was conducted by Kips et al., who demonstrated that reslizumab reduced both sputum and blood eosinophil counts, as well as transiently increased FEV_1_ (116). A further phase II study, performed by Castro et al. in patients with severe eosinophilic asthma, showed that reslizumab induced a significant FEV_1_ increase, associated with a non-significant tendency towards an improvement in asthma control, particularly in asthmatic subjects with high blood eosinophil numbers and coexistent nasal polyposis (117). Subsequently, two phase III trials carried out by Castro et al. in patients with severe asthma and blood eosinophil counts higher than 400 cells/μL, evidenced that reslizumab lowered the annual asthma exacerbation rate by more than 50%, improved asthma control and incremented FEV_1_ (118). Such findings were further confirmed by Brusselle et al., especially in subjects with late-onset eosinophilic asthma (119). Moreover, Bjermer et al. observed that the positive effects of reslizumab on lung function were not limited to the large airways, as shown by FEV_1_ increases, but also extended to the small airways resulting in significant enhancements of mid-expiratory flow at 25–75% of forced vital capacity (FEF_25-75_) (120). Similar to omalizumab and mepolizumab, also reslizumab displays a more than satisfactory profile of safety and tolerability (114).

Differently from mepolizumab and reslizumab, benralizumab is characterized by a dual mechanism of action. Indeed, this humanized afucosylated IgG1/κ monoclonal antibody of murine origin binds through its Fab fragments to IL-5Rα, thereby impeding the assembly of the ternary molecular complex consisting of IL-5, IL-5Rα, and the βc subunits of IL-5 receptor (121, 122); as a consequence, IL-5 cannot exert its biological effects on target cells (eosinophils, basophils, ILC2). Moreover, *via* the constant Fc portion benralizumab interacts with the surface FcγRIIIa receptor of natural killer cells, thus triggering eosinophil apoptosis operated by antibody-dependent cell-mediated cytotoxicity (ADCC), a mechanism that is remarkably potentiated by afucosylation (121, 122). In regard to the randomized clinical trials, phase III SIROCCO and CALIMA studies have demonstrated that benralizumab significantly reduced the annual rate of severe eosinophilic asthma exacerbations, and also bettered asthma symptom control and increased FEV_1_ (123, 124). Chipps et al. performed a pooled analysis of SIROCCO and CALIMA trials, thus showing that benralizumab was effective as adjunctive biological therapy in both allergic and non-allergic patients with severe eosinophilic asthma (125). The BISE trial confirmed the positive impact of benralizumab on lung function, whereas the ZONDA study showed that benralizumab significantly decreased daily OCS intake (126, 127). Furthermore, the BORA trial documented that benralizumab use was associated with long-term safety and tolerability (128). All these findings, regarding clinical and functional outcomes, have been corroborated, and even extended and amplified by recent real-life experiences. The latter are providing convincing evidence referring to the safety and efficacy of benralizumab, detected in both atopic and non-atopic subjects with eosinophilic uncontrolled asthma, with regard to relevant improvements in asthma exacerbations, OCS consumption, symptom control, airflow limitation, lung hyperinflation, and nasal polyposis (129–132).

Dupilumab is a fully human IgG4 monoclonal antibody, which specifically recognizes and occupies the α subunit of IL-4 receptor, thereby inhibiting the biological actions of both IL-4 and IL-13 (133). Indeed, these two cytokines not only exert overlapping effects related to IgE class switching, eosinophil chemotaxis and airway hyperresponsiveness, but also share common receptor mechanisms and signalling pathways, based on activation of IL-4Rα coupled to the JAK/TYK transduction machinery (10, 133). Therefore, dupilumab behaves as a dual receptor antagonist of IL-4 and IL-13 (134). In an initial phase IIa trial, Wenzel et al. randomly assigned to either dupilumab or placebo 104 patients with persistent, moderate-to-severe eosinophilic asthma, thus showing that dupilumab significantly lowered the asthma exacerbation rate by 87%, and also enhanced FEV_1_ by more than 200 mL, despite ICS/LABA withdrawal (135). A subsequent larger, phase IIb study carried out in uncontrolled adult asthmatics confirmed the positive impact of dupilumab on asthma exacerbations and lung function, especially but not only in subjects with high blood eosinophil counts (136). More recently, the phase III LIBERTY ASTHMA QUEST trial showed that in asthmatic patients with blood eosinophil numbers ≥ 300 cells/μL, dupilumab was able to decrease asthma exacerbations by more than 65%, as well as to increase FEV_1_ by more than 200 mL (137). Corren et al. performed a *post hoc* analysis of the LIBERTY ASTHMA QUEST study, thereby demonstrating that the above beneficial effects of dupilumab can be indifferently detected in both allergic and non-allergic asthmatics (138). Furthermore, the LIBERTY ASTHMA VENTURE trial highlighted the significant OCS-sparing action of dupilumab (139). Overall, dupilumab is quite safe and well tolerated, even if in some patients this biologic drug can induce conjunctivitis or a marked blood eosinophilia (10), which however tends to resolve spontaneously in a few months, without apparent consequences in most cases. Dupilumab is also very effective for treatment of relevant asthma comorbidities such as atopic dermatitis and nasal polyposis (140, 141).

The molecular targets, mechanisms of action and therapeutic effects of the above mentioned drugs are summarized in **Table 1**.

**Table 1:** Licensed biological therapies for severe asthma.

Licensed biological therapies	Targets	Molecular mechanisms of action	Effects in the control of severe asthma
Omalizumab	IgE	Generation of IgE/anti-IgE immune complexes that inhibit IgE-mediated allergic cascade	↓ Exacerbations ↑ Quality of life and symptom control ↑ FEV1
Mepolizumab	IL-5	Prevention of IL-5 binding to IL-5Rα	↓ Blood and sputum eosinophils ↓ Exacerbations ↑ Quality of life and symptom control ↓ OCS intake ↑ FEV1
Reslizumab	IL-5	Prevention of IL-5 binding to IL-5Rα	↓ Blood and sputum eosinophils ↓ Exacerbations ↑ Quality of life and symptom control ↑ FEV1
Benralizumab	IL-5Rα	Blockade of IL-5Rα ADCC-induced eosinophil apoptosis	↓ Blood eosinophils ↓ Exacerbations ↑ Quality of life and symptom control ↓ OCS intake ↑ FEV1
Dupilumab	IL-4Rα	Dual receptor antagonism of IL-4/IL-13	↓ Exacerbations ↓ OCS intake ↑ FEV1

## Targets of Emerging Biological Therapies in Clinical Development

In addition to the currently available biological therapies of severe asthma, the recent advances in our understanding of the pathobiology of this complex disease are allowing to disclose new potential targets for future anti-asthma treatments. In particular, besides the downstream effectors of type 2 airway inflammation such as IgE, IL-5, IL-4/13 and their receptors, other very interesting pathogenic molecules include upstream activators of cellular pathways leading to T2-high asthma. Within this context, a key role is played by the innate cytokines known as alarmins, including TSLP, IL-33, and IL-25 (142). So far, the most extensively studied alarmin as suitable target for novel biological therapies of asthma has been TSLP (13, 143).

TSLP bioactivities are involved in several pathogenic aspects of type 2 asthma. Indeed, by up-regulating OX40 ligand expression, TSLP acts as a powerful inducer of dendritic cell activation (144). Upon TSLP-mediated stimulation, dendritic cells drive naïve Th lymphocytes towards differentiation into active Th2 cells producing IL-4, IL-5, and IL-13 (145). Moreover, TSLP up-regulates the expression of such cytokines at level of other cellular sources, including basophils, mast cells, and especially ILC2 (142, 146, 147). In regard to these latter cells, TSLP also promotes their survival and steroid resistance (147, 148). TSLP appears to be also implicated in T2-low asthma pathobiology. In fact, this alarmin can induce dendritic cells to drive the commitment of naïve Th cells towards a Th17 immunophenotype (149).

Tezepelumab is an anti-TSLP fully human monoclonal antibody (**Figure 1**), which prevents TSLP binding to its receptor complex (150). Tezepelumab was firstly tested in patients with mild allergic asthma by Gauvreau et al., who noted that this anti-TSLP antibody reduced allergen-induced FEV_1_ decreases, as well as post-allergen increases in blood/sputum eosinophils and FeNO (151). A subsequent phase IIb trial was carried out by Corren et al., who showed that tezepelumab decreased the annualized asthma exacerbation rate by 60–70% and enhanced pre-bronchodilator FEV_1_, independently of blood eosinophil numbers (152). Furthermore, tezepelumab lowered the most relevant biomarkers of T2-high asthma, including IgE serum concentrations, blood eosinophil counts and FeNO levels (152). Ongoing phase II and III trials are evaluating the safety profile of tezepelumab, as well as its eventual efficacy in decreasing airway inflammation and OCS intake (142). So far, tezepelumab has not yet been investigated in regard to its potential therapeutic effects in patients with T2-low asthma.

IL-33 cooperates with TSLP in promoting type-2 immune/inflammatory responses (153). In particular, IL-33 induces airway hyperresponsiveness by stimulating IL-13 release from ILC2 and mast cells (154, 155). Several phase II trials are underway with the aim of evaluating some biologic drugs which target IL-33 or its ST2 receptor (142). In particular, it has been shown that the anti-IL-33 monoclonal antibody REGN3500 (**Figure 1**) was able to improve the control of severe asthma, but its therapeutic effects did not result to be better than those induced by dupilumab (142). Moreover, when tested in association with this IL-4/IL-13 dual receptor antagonist, the anti-asthma actions of such two biologicals were comparable to those exerted by dupilumab alone (142).

Although IL-25 plays a relevant pathogenic role in allergic inflammation, to our knowledge no anti-IL-25 monoclonal antibody is currently in clinical development for add-on treatment of severe asthma.

Another key mediator of type-2 asthma is prostaglandin D_2_ (PGD_2_), mainly produced by mast cells (156). PGD_2_ exerts its pro-inflammatory actions *via* stimulation of CRTH2 (chemoattractant receptor-homologous molecule expressed on Th2 cells) receptor, expressed by Th2 lymphocytes, ILC2 and eosinophils (156). Binding of PGD_2_ to CRTH2 can be blocked by fevipiprant (**Figure 1**), a selective receptor antagonist which is not a monoclonal antibody, but rather a small compound used as an oral drug (156). Despite the partially promising results of some preliminary studies carried out in asthmatic patients, however fevipiprant seems to induce only a weak FEV_1_ increase, similar to the functional effect of the leukotriene receptor antagonist montelukast (157, 158). Further studies are thus needed, even if the therapeutic potential of fevipiprant for asthma therapy currently appears to be quite uncertain.

The molecular targets, mechanisms of action and therapeutic effects of the above mentioned drugs are summarized in **Table 2**.

**Table 2:** New potential targets of emerging anti-asthma therapies.

New potential targets	New potential drugs	Molecular mechanisms of action	Effects in the control of severe asthma
TSLP	Tezepelumab	Prevention of TSLP binding to its receptor complex	↓ Blood eosinophils ↓ FeNO ↓ Exacerbations ↑ FEV1
IL-33	REGN3500	Prevention of IL-33 binding to ST2 receptor	↑ Quality of life and symptom control
PGD2	Fevipiprant	Selective antagonism of CRTH2 receptor	Weak FEV1 increase

With regard to the potential molecular targets of biological therapies for type 2-low neutrophilic asthma, the main focus of current studies is the pathogenic axis connecting IL-1β, IL-23, and IL-17. In particular, the IL-1 receptor antagonist anakinra and the anti-IL-1β monoclonal antibody canakinumab are currently under clinical investigation in phase I/II trials enrolling patients with mild asthma (159, 160). In addition, further phase II studies are evaluating, in patients with severe type 2-low asthma, the efficacy and safety of the anti-IL-23 antibody risankizumab, as well as of an anti-IL-17A monoclonal antibody (159, 160). However, a previous trial carried out in moderate-to-severe asthmatics, aimed to investigate the effects of the anti-IL-17 receptor monoclonal antibody brodalumab, did not show any improvement in asthma symptom control and lung function (161).

## Conclusions

Ongoing progress in our knowledge of the pathobiological mechanisms underlying the various cellular and molecular phenotypes of severe asthma has made it possible to unveil suitable targets for add-on biological therapies. Several approved anti-IgE, anti-IL-5, anti-IL-5 receptor, and anti-IL-4/IL-13 receptor monoclonal antibodies are currently prescribed by clinicians. These drugs are helping patients with severe, allergic or non-allergic eosinophilic T2-high asthma, to significantly improve symptom control, lung function and global health status, and especially to lessen their susceptibility to suffer from frequent and often serious disease exacerbations. Moreover, new promising monoclonal antibodies, mainly targeting the innate cytokines known as alarmins, are in advanced clinical development. However, patients with severe T2-low asthma are largely excluded from the therapeutic benefits achievable by people who experience T2-high severe disease. Therefore, in the coming years strong research efforts should be finalized to develop novel biological treatments for severe neutrophilic or paucigranulocytic asthma, thus hoping that patients expressing such uncontrolled phenotypes may pursue in the near future better health conditions than the current ones.

## Author Contributions

All authors contributed to elaborate the text and to draw the figure. All authors contributed to the article and approved the submitted version.

## Conflict of Interest

The authors declare that the research was conducted in the absence of any commercial or financial relationships that could be construed as a potential conflict of interest.
